# Effect of Cell Spreading on Rosette Formation by Human Pluripotent Stem Cell-Derived Neural Progenitor Cells

**DOI:** 10.3389/fcell.2020.588941

**Published:** 2020-10-15

**Authors:** Ryan F. Townshend, Yue Shao, Sicong Wang, Chari L. Cortez, Sajedeh Nasr Esfahani, Jason R. Spence, K. Sue O’Shea, Jianping Fu, Deborah L. Gumucio, Kenichiro Taniguchi

**Affiliations:** ^1^Department of Cell & Developmental Biology, University of Michigan Medical School, Ann Arbor, MI, United States; ^2^Department of Mechanical Engineering, University of Michigan, Ann Arbor, MI, United States; ^3^Department of Internal Medicine, University of Michigan Medical School, Ann Arbor, MI, United States; ^4^Department of Cell Biology, Neurobiology, and Anatomy, Medical College of Wisconsin, Milwaukee, WI, United States; ^5^Department of Pediatrics, Medical College of Wisconsin, Milwaukee, WI, United States

**Keywords:** neural rosette, human pluripotent stem cells, cell spreading, neural progenitor cells, actin cytoskeletal network, RhoA, neural tube, microcontact printing

## Abstract

Neural rosettes (NPC rosettes) are radially arranged groups of cells surrounding a central lumen that arise stochastically in monolayer cultures of human pluripotent stem cell (hPSC)-derived neural progenitor cells (NPC). Since NPC rosette formation is thought to mimic cell behavior in the early neural tube, these rosettes represent important *in vitro* models for the study of neural tube morphogenesis. However, using current protocols, NPC rosette formation is not synchronized and results are inconsistent among different hPSC lines, hindering quantitative mechanistic analyses and challenging live cell imaging. Here, we report a rapid and robust protocol to induce rosette formation within 6 h after evenly-sized “colonies” of NPC are generated through physical cutting of uniformly polarized NESTIN^+^/PAX6^+^/PAX3^+^/DACH1^+^ NPC monolayers. These NPC rosettes show apically polarized lumens studded with primary cilia. Using this assay, we demonstrate reduced lumenal size in the absence of *PODXL*, an important apical determinant recently identified as a candidate gene for juvenile Parkinsonism. Interestingly, time lapse imaging reveals that, in addition to radial organization and apical lumen formation, cells within cut NPC colonies initiate rapid basally-driven spreading. Further, using chemical, genetic and biomechanical tools, we show that NPC rosette morphogenesis requires this basal spreading activity and that spreading is tightly regulated by Rho/ROCK signaling. This robust and quantitative NPC rosette platform provides a sensitive system for the further investigation of cellular and molecular mechanisms underlying NPC rosette morphogenesis.

## Introduction

The radial organization of cells into rosette-like structures containing a central apical lumen is a fundamental developmental hallmark of neuroepithelial tissue (Tomooka et al., 1993; Deglincerti et al., 2016). Recent methodological advances in the directed differentiation of human pluripotent stem cells (hPSC) have resulted in a variety of neural differentiation methods to generate monolayers of apico-basally polarized neural stem cell (NPC) types (Chambers et al., 2009; Shi et al., 2012; Lukovic et al., 2017). Interestingly, in some neural differentiation culture systems, NPC monolayers undergo spontaneous radial morphogenesis and apical constriction, forming NPC rosettes, or neural rosettes, which are apico-basally polarized pseudostratified structures similar to the embryonic neural tube (Haigo et al., 2003; Curchoe et al., 2012; Christodoulou and Skourides, 2015; Deglincerti et al., 2016; Nikolopoulou et al., 2017).

Rosette formation from NPC *in vitro* provides an important model to study the underpinnings of normal and defective human neural tube morphogenesis and defects in this *in vitro* process have been documented in several neurological disorders, including bipolar disorder, autism spectrum disorder and schizophrenia (Ladran et al., 2013; Chen et al., 2014; Kim et al., 2014; Yoon et al., 2014; Madison et al., 2015; O’Shea and McInnis, 2016; Valensisi et al., 2017). Recently, abnormal rosette formation was demonstrated in patients with schizophrenia and traced to haploinsufficiency of CYFIP1, a WAVE complex component that regulates Arp2/3 and controls apico-basal polarity (Yoon et al., 2014). Additionally, deletion of *Crb2*, also a cell polarity protein, in mouse NPC results in defective neural rosette formation, even though *Crb2*^–/–^ NPC monolayers show normal polarization (Boroviak and Rashbass, 2011), emphasizing that rosette structures have apico-basal properties that are distinct from those of polarized monolayers. Interestingly, recent genetic studies implicate *Podocalyxin like* (*PODXL*), a gene encoding a sialyated apical glycoprotein that is highly expressed in neural tissue, as a causal gene in autosomal-recessive juvenile onset Parkinsonism (Sudhaman et al., 2016). PODXL has been well studied in early mouse embryogenesis and in kidney for its role in apical polarization and lumen formation (Takeda et al., 2000; Bryant et al., 2014; Yang et al., 2016; Shahbazi et al., 2017; Christodoulou et al., 2018), but the role of this protein in rosette formation has not been reported.

Although NPC rosette formation *in vitro* can be a powerful tool for detection and investigation of molecular pathways important in human neurological disease, a major limitation to current methods for rosette generation is that rosettes arise in a sporadic and uncoordinated manner. This makes it difficult to reproducibly, systematically and quantitatively study the molecular and cellular requirements of this process using live-imaging, biomechanical platforms, small molecule screens and/or genetic manipulation. Finally, while current assays can measure attributes of individual mature rosettes, a complete mechanistic understanding of the dynamics of NPC rosette morphogenesis, starting from a polarized monolayer and ending in a fully-formed rosette, is currently limited.

Here, we report a protocol that efficiently induces near synchronous rosette organization, even from hPSC-derived NPC monolayers that do not generally exhibit rosette formation using previously existing protocols. Robust radial organization is seen within 6 h after induction; this rapid response lends itself to mechanistic and quantitative analyses. Using this assay, we document lumenal size defects in NPC carrying loss-of-function mutations of *PODXL*, a feature that may have implications for early onset Parkinson’s disease caused by loss of PODXL activity (Sudhaman et al., 2016). Further, we trace the cellular dynamics of NPC rosette formation using live cell imaging. These data reveal that an early aspect of rosette formation is dynamic spreading of the basal portions of cells within NPC colonies. Using chemical, genetic and engineering tools, we show that this basal colony spreading is required for successful NPC rosette morphogenesis. Thus, this novel NPC rosette induction system enables robust mechanistic analyses that reveal new cellular and molecular details about NPC rosette formation and polarization. Such a reproducible assay is critical requirement for quantitative examination of alterations in rosette formation that might accompany a variety of human diseases and could be diagnosed using induced pluripotent stem cells (hiPSC).

## Results

### Development of a Simple and Robust Neural Differentiation Protocol in an Extracellular Matrix-Based, Feeder-Free Condition

**FIGURE 1:**
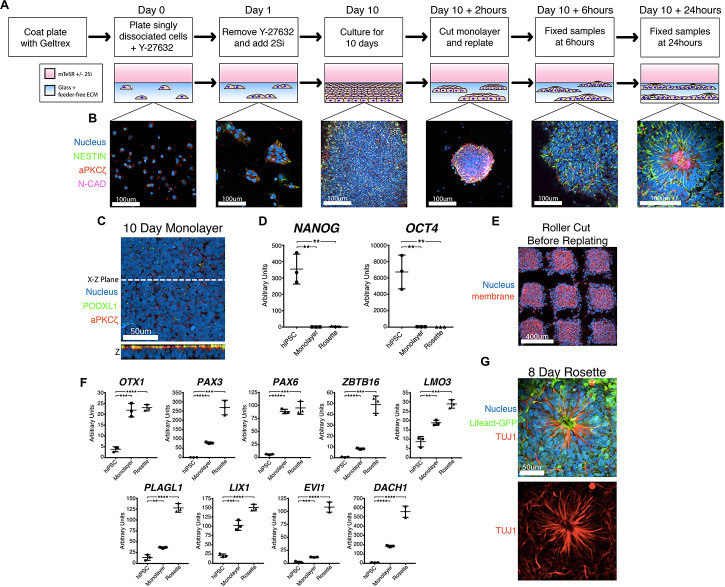
A novel robust and quantitative method for NPC rosette induction. **(A,B)** Overview of the NPC rosette induction protocol, using a Geltrex-based feeder-free ECM substrate and mTeSR medium with dual SMAD inhibitors (2Si). Singly dissociated cells were grown for 10 days to allow the formation of a tightly packed NPC monolayer. At d10, the NPC monolayer was cut into small pieces, scraped off the substrate and re-plated to allow the formation of NPC rosettes. Cells were stained with antibodies to NESTIN (green), aPKCξ (red) and N-CAD (purple). DAPI = Nuclei (Blue). **(C)** Apical surface of the d10 NPC monolayer. An optical section along X–Z-plane reveals that the monolayer formed a uniformly polarized apical membrane. Apical markers: PODXL (green) and aPKCξ (red). **(D)** Relative expression of pluripotency markers (*NANOG* and *OCT4*) as determined by quantitative PCR (qPCR). hiPSC = 1196a hiPSC monolayer (no neural induction), Monolayer = d10 NPC monolayer, and Rosette = NPC rosettes, 24 h after cutting and re-plating. RNA expression is normalized (to *GAPDH*); mean and standard deviation (SD) is shown. **(E)** Confocal section of the roller-cut d10 NPC monolayer, stained with DAPI (Nucleus) and WGA (membrane), revealing colonies with highly consistent sizes. **(F)** qPCR analysis of neural markers: *OTX1*, *PAX3*, *PAX6*, *ZBTB16*, *LMO3*, *PLAGL1*, *LIX1*, *EVI1*, *DACH1*. For all qPCR analyses: hiPSC = 1196a hiPSC monolayer (no neural induction), Monolayer = d10 NPC monolayer, and Rosette = NPC rosettes 24 h after cutting and re-plating. Relative RNA expression is normalized (to *GAPDH*); mean and standard deviation (SD) is shown for all qPCR analyses. **(G)** Immunolocalization of TUJ1, a neuron-specific Class III γ-tubulin, in a rosette generated from cells carrying Lifeact-GFP, cultured for 8 days after roller-dissociation. Student’s *t*-test was used for all statistical analyses: ns = *p* > 0.05; **p ≤ 0.01; ***p ≤ 0.001; ****p ≤ 0.0001.

Multiple protocols exist for the generation of NPC rosettes (Dhara and Stice, 2008; Chambers et al., 2009; Shi et al., 2012; Knight et al., 2018); these differ in multiple aspects (e.g., 3D vs. 2D, presence or absence of feeder cells, differing growth media). We employed the dual SMAD inhibitor-based NPC monolayer induction protocol (using 2Si, SB-431542 and LDN-193189 to inhibit TGFβ and BMP receptor kinase activity, respectively) originally developed by Chambers et al. (2009), rather than another commonly utilized embryoid body system that has inherent heterogeneity and also gives rise to non-neural cell types (Zhang et al., 2001; Reinhardt et al., 2013). We utilized a feeder-free substrate coated with Geltrex extracellular matrix, since this substrate is routinely used to culture a variety of hPSC lines (Figure 1A; Shao et al., 2017a; Shao et al., 2017b; Taniguchi et al., 2017). While a laminin-511 feeder-free substrate had previously been shown to support neural differentiation (Nakagawa et al., 2014), the Geltrex feeder-free system had not been previously tested. This 2Si-based neural differentiation was performed using mTeSR medium to provide additional pro-neural factors (such as FGF2 and LiCl, (Taupin et al., 2000; Elkabetz et al., 2008; Qi et al., 2017)). This culture method results in the formation of a dense apicobasally polarized monolayer of neural cells that exhibit expression of neural markers, NESTIN and N-CADHERIN (Figures 1B,C) as well as significantly reduced expression of *OCT4* (also known as *POU5F1*) and *NANOG* within 10 days (Figure 1D). Thus, this Geltrex feeder-free system, in combination with 2Si and mTeSR, can be utilized to support efficient differentiation of hPSC toward the neural lineage.

### Development of a Robust NPC Rosette Formation System

Using this mTeSR/2Si neural differentiation method, NPC monolayers generated from H9 hESC exhibit abundant rosette morphogenesis (Supplementary Figure 1A, H9 hESC 10 days) as previously seen in other 2Si culture conditions using Essential 6 (E6)/2Si medium (Lippmann et al., 2014). However, two tested hiPSC lines (1196a – Figures 1B,C, Day 10; and iPSC20-1 – Supplementary Figure 1A, iPSC20-1 10 days) failed to form radially organized colonies under mTeSR/2Si or E6/2Si conditions (Supplementary Figure 1B). Others have reported that dissociation of NPC monolayers by manual scraping and re-plating the cells as small clumps or “colonies” induces radial patterning using hESC and hiPSC lines (Shi et al., 2012). The culture conditions previously described by Shi et al. (2012) readily induced formation of radially patterned structures when H9 hESC were manually scraped, and re-plated as small clumps (Supplementary Figure 1C, top, H9 hESC 24-hour colony). However, when using 1196a hiPSC, rosette morphogenesis was rarely seen with the (Shi et al., 2012) protocol (Supplementary Figure 1C, bottom, see 1196a 24-hour colony), demonstrating cell line-specific variability of this system to induce rosette morphogenesis.

Interestingly, when 2Si/mTeSR conditions were used and NPC monolayers derived from 1196a and iPSC20-1 were manually dissociated and re-plated as small colonies (manual-dissociation), rosette formation was seen in the majority (69.44% ± 1.338, *n* = 360) of the colonies (Supplementary Figure 1A, 1196a and iPSC20-1 24-hour colony). However, such manually dissociated colonies were highly variable in size (Supplementary Figure 1D, scraped), preventing reproducible quantitative analyses. To generate colonies with more consistent sizes, we used a roller-based StemPro EZPassage Disposable Stem Cell Passaging Tool (Thermo-Fisher, schematics of this tool shown in Supplementary Figure 1E) to roller-cut NPC monolayers (roller-dissociation) into approximately 0.022 mm^2^ ± 0.003 SD (*N* = 35) colonies (Figure 1E and Supplementary Figure 1D, see Roller Cut, and S1F for size quantitation). After attachment for 1 h, colony size variation was significantly reduced, compared to the manual dissociation method (Supplementary Figure 1F, *F*-test: *p* < 0.01): colony size at the 1-hour time-point was 0.016 mm^2^ ± 0.007 SD (*N* = 38) in size for roller cut and 0.010 mm^2^± 0.016 SD (*N* = 68) for manually scraped cells.

Radial morphogenesis of roller-cut NPC colonies is very rapid; rosettes can be observed as early as 6 h after plating cut colonies (Figure 1B, see Day 10 +6 h, see Supplementary Figure 1G for a large field-of-view image). Mature rosettes are formed by 24 h (Figure 1B, Day 10 +24 h). Importantly, cutting alone is not sufficient to induce radial morphogenesis, since no signs of radial organization are seen in 12-hr colonies that were roller-cut, but not replated (Supplementary Figure 1H). NPC rosettes exhibit expression of several markers of neural differentiation, including *OTX1, PAX3, PAX6, ZBTB16, LMO3, PLAGL1, LIX1, EVI1*, and *DACH1* (Figure 1F; Elkabetz et al., 2008; Reinhardt et al., 2013; Deglincerti et al., 2016). Within 7 days of extended culture, cells within the NPC rosettes display a highly elongated morphology with processes that extend beyond the radial core; in fact, these cells express TUJ1, neuron-specific Class III γ-tubulin (Figure 1G, see Supplementary Figure 1I for large-field of view). Thus, combined with roller-dissociation, this 2Si/mTeSR-based system reproducibly triggers rapid and robust generation of NPC rosette formation as well as subsequent differentiation of TUJ1^+^ neurons from hPSC lines that do not show radially organized phenotypes using existing protocols.

We next tested the ability of this system to effectively detect aberrancies in rosette formation after exposure of cells to compounds known to affect rosette morphogenesis. Yoon et al. (2014) previously showed that *CYFIP1*, a component of the WAVE complex, is critical for NPC rosette formation through its regulation of Arp2/3-dependent actin polymerization. Thus, we allowed roller cut colonies to attach for 2 h and then treated them with CK-666, a small molecule inhibitor of Arp2/3, for 10 h. Indeed, this relatively short exposure of cells to the inhibitor significantly altered rosette formation, reducing total NPC aPKCζ^+^ lumenal area (∼3.4-fold reduction, *p* < 0.1, Supplementary Figures 2A–C) as well as radial organization, as measured by nuclear aspect ratio (1.44-fold reduction, *p* < 0.01, Supplementary Figure 2D). However, colony size and number of lumens per colony do not significantly change with CK-666 treatment (Supplementary Figures 2E,F). Thus, the system is sensitive to rosette perturbation by small molecule inhibitors of actin polymerization.

### Characteristics of the Apical Domain of Roller-Dissociated NPC Rosettes

**FIGURE 2:**
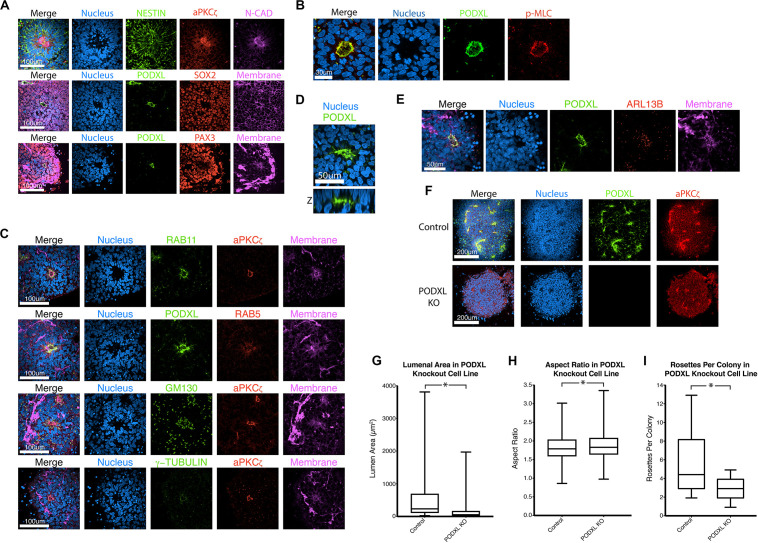
Characterization of NPC rosettes. **(A–E)** Immunolocalization analyses of NPC rosettes with indicated markers: neural markers = NESTIN, N-CADHERIN (N-CAD), SOX2 and PAX3; apical markers = aPKCζ and PODXL; myosin activity marker = p-MLC; and organelle markers = RAB11 (recycling endosome), RAB5 (early endosome), GM130 (Golgi), γ-TUBULIN (centrosome) and ARL13B (primary cilium). In **(D)**, the X-Z plane is shown to reveal the internalized PODXL^+^ apical domain. Note that the rosette that is stained for NESTIN, aPKCζ, N-CAD and DNA is identical to the Day 10 + 24 hr sample in Figure 1B. **(F)** Confocal fluorescent images of control and *PODXL*-KO NPC rosettes stained with indicated markers. **(G–I)** Quantitation of lumenal area **(G)**, aspect ratio **(H)** and number of rosettes per colony **(I)** of control and *PODXL*-KO cells. Student’s *t*-test was used for statistical analyses: ns = *p* > 0.05; *p ≤ 0.05.

Neural progenitor cell rosettes are described as polarized structures with a central apically constricted domain surrounded by radially organized cells (Boroviak and Rashbass, 2011; Deglincerti et al., 2016); these features are also seen in roller-dissociated 2Si/mTeSR NPC rosettes (Figures 2A,B). Additional immunolocalization analyses reveal that recycling (RAB11) and early (RAB5) endosomes are localized adjacent to the apical cortex, and Golgi (GM130) and centrosomes (γ-TUBULIN) are localized at the apical pole of the cells (Figure 2C), further confirming the apical nature of the central domain. Interestingly, 3D reconstruction of NPC rosettes stained for the apical marker PODXL reveals that cells completely surround the lumen (Figure 2D), confirming recent studies (Hribkova et al., 2018). Indeed, the apical region of each cell is studded with a single primary cilium (ARL13B, Figure 2E); this is in accord with the localization of the centrosomes (γ-TUBULIN, Figure 2C). This polarized organization of organelles is topologically similar to *in vitro* lumenal cyst models documented in cell types such as MDCK.2, Caco-2, and hPSC (Rodriguez-Boulan and Macara, 2014; Taniguchi et al., 2015) that show apical localization of endosomes, Golgi, centrosomes and cilia.

In other systems of apical polarization, it has been demonstrated that PODXL is necessary for apical actin cytoskeletal organization and lumen expansion (Takeda et al., 2000; Bryant et al., 2014; Shahbazi et al., 2017; Christodoulou et al., 2018). To test this requirement in the rosette system, we generated a loss-of-function mutation in *PODXL* in 1196a hiPSC (Figures 2F–H, see Supplementary Figure 3 for genotyping information) and examined rosette formation using two independent lines of successfully targeted cells. Quantitation shows that, 12 h after plating, *PODXL* mutant rosettes exhibit fewer rosettes/colony and reduced lumenal sizes (quantified using aPKCζ^+^ area as shown in Supplementary Figure 2B) while exhibiting largely intact radial organization (Figures 2G–I). Thus, *PODXL*, which is abundant in the developing brain (Nowakowski et al., 2010; Vitureira et al., 2010; Sudhaman et al., 2016), is important for lumenal initiation and expansion in neural rosettes.

### Colony Dynamics During NPC Rosette Formation

**FIGURE 3:**
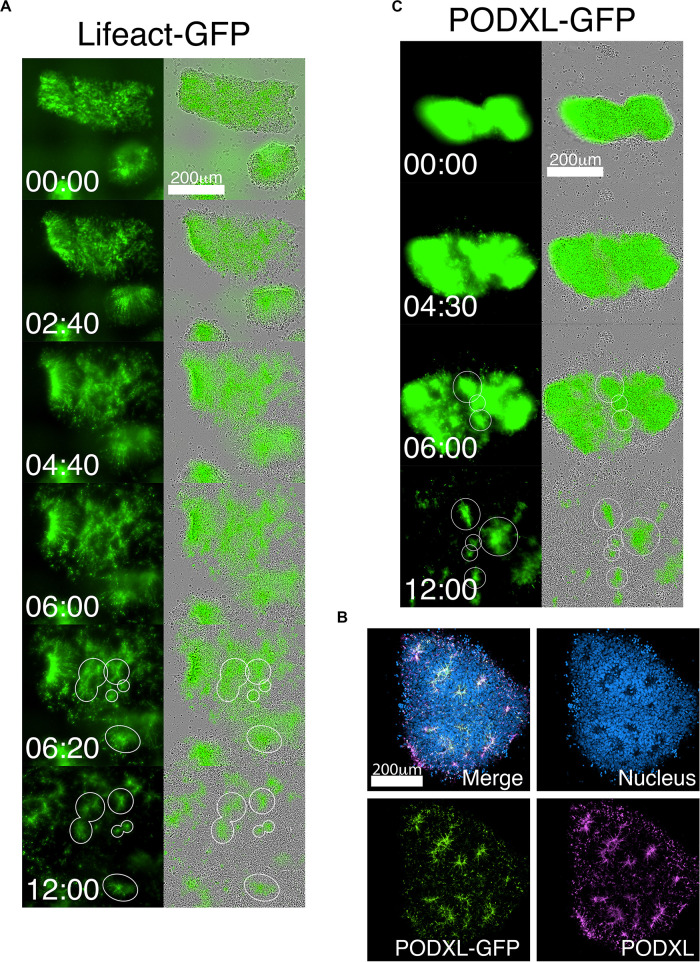
Live-tracking of NPC rosette morphogenesis. **(A–C)** Live imaging of 1196a hiPSC expressing Lifeact-GFP **(A)** or PODXL-GFP **(C)** for 12 h after colonies were allowed to reattach for 2 h. **(B)** Confocal images of PODXL-GFP NPC rosettes stained with anti-PODXL. GFP localization mirrors endogenous PODXL localization. Note that, while a uniform prominent GFP signal is initially seen throughout the apical surface of the recently plated colony, at later time points, GFP-enriched regions are concentrated at the constricted centers of the rosettes (lumen). It is likely that, as the colony expands rapidly, the apical surface area of non-rosette cells dramatically increases, resulting in reduced GFP signal intensity per unit area. Scales as indicated.

The robust and rapid nature of NPC rosette formation, as well as consistent colony size produced by roller-dissociation of the NPC monolayer, allowed us to perform quantitative live imaging to investigate the morphogenic steps that characterize the formation of NPC rosettes. We utilized live tracking of hiPSC, stably expressing Lifeact (live-actin)- or PODXL-GFP fusion proteins (Figures 3A–C; Taniguchi et al., 2017). F-actin (visualized using Lifeact-GFP) is initially localized uniformly across the entire apical surface of recently cut NPC colonies. However, by 6 h after plating, localized apical constriction produces multiple Lifeact-GFP foci (rosette lumens), surrounded by radially organized NPC (Figure 3A and Supplementary Movie 1, see 06:20 and 12:00).

As a more specific label of the apical domain, we next utilized PODXL-GFP (Figures 3B,C and Supplementary Movie 2). Again, freshly cut colonies show abundant fluorescence across the apical surface of all cells, consistent with the fact that the initial NPC monolayer is uniformly apically polarized (Figure 1C). However, within hours after plating the cut colonies, PODXL-GFP is localized in multiple distinct foci that are shared by groups of radially oriented cells (Figure 3C and Supplementary Movie 2). Additionally, while some rosettes continue radial organization, others undergo previously unreported progressive fission of established radial domains to give rise to secondary NPC rosettes (Supplementary Movie 2 center, see 9:00 to 11:00). Together, these data reveal a dramatic re-organization of the apical domain as rosettes are formed.

### Basal Cell Spreading Is an Early Feature of NPC Rosette Organization

**FIGURE 4:**
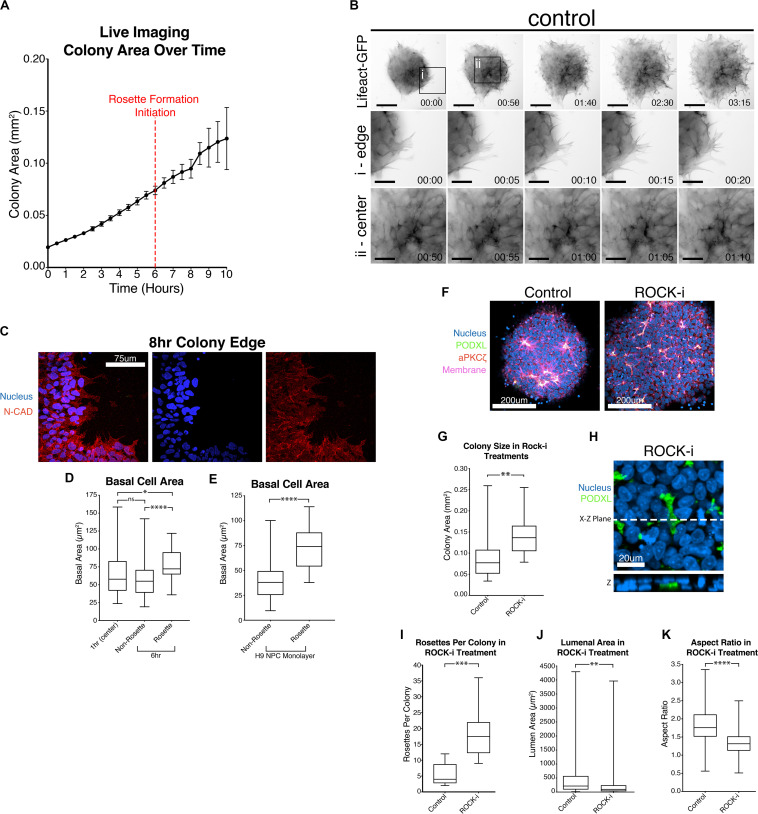
Colony spreading regulates NPC rosette formation. **(A)** Quantitation of the growth of PODXL-GFP colonies over time. Imaging initiated 2 h after plating. Red dotted line at the 6-hr time point (8 h after plating) indicates the start of rosette morphogenesis. **(B)** Live imaging of 1196a hiPSC expressing Lifeact-GFP 2 h after roller cutting and re-plating. Imaging was started 5 h after re-plating. Top panels show images of whole colonies. (i) and (ii) panels show magnified images at the colony edge (i) or at the center (ii). Scales indicate 150 μm for whole colony, 50 μm for magnified images in i and ii. **(C)** Confocal images of 8 h NPC rosette colony edge cells stained with indicated markers. **(D)** Quantitation of basal cell area (based on N-CADHERIN membrane staining) of roller-dissociated 1196a hiPSC-derived NPC colonies 1 h after plating at the center (1 h (center)) and 6 h after plating in cells that are inside (Rosette) and outside (Non-Rosette) of rosettes. **(E)** Quantitation of basal cell area of H9 hESC-derived day 10 NPC monolayer (no roller-cut) in cells that are inside (Rosette) and outside (Non-Rosette) of rosettes. **(F)** Confocal images of NPC rosettes treated with DMSO (control) or ROCK-i, stained with indicated markers. **(G)** Quantitation of colony size from samples in panel **(F)**. **(H)** Immunolocalization analysis of PODXL^+^ domain in ROCK-i treated samples: the X–Z plane is shown to reveal the internalized PODXL^+^ apical domain. **(I–K)** Quantitation of number of rosettes per colony **(I)**, individual lumen area **(J)** and nuclear aspect ratio **(K)** from samples in panel **(F)**. Scales as indicated. Student’s *t*-test was used for statistical analyses: *p ≤ 0.05; **p ≤ 0.01; ***p ≤ 0.001; ***p < 0.0001.

Interestingly, live imaging using both Lifeact-GFP and PODXL-GFP reveals that cut NPC colonies quickly increase in size as soon as they attach to the substrate (Figures 3A,C and Supplementary Movies 1, 2). This colony expansion precedes radial organization and continues during radial morphogenesis. Detailed quantitation shows that these colonies expand 4-fold in size by 8 h after plating (6-hour timepoint in Figure 4A, live imaging initiated 2 h after plating, red dotted line) and 6-fold by 12 h (Figure 4A).

Given this unexpected finding, cell expansion was further examined at the cellular level using hiPSC stably expressing Lifeact-GFP, 5 h after plating, when colonies had attached and were beginning to initiate radial organization. Under these conditions, continuous colony spreading is seen during 200 min of imaging, at 5-minute intervals (Figure 4B, control, and Supplementary Movies 3–5). At the cellular level, two types of spreading are detectable. Cells at the edge of the colony exhibit dynamic protrusion and retraction of filopodia and lamellipodia, visible by Lifeact staining of these organized actin-rich protrusions (Figure 4Bi, control, Supplementary Movie 4). Despite the increased basal protrusive activity, these edge cells maintain cell membrane localization of N-CADHERIN directly adjacent to neighboring cells (Figure 4C), indicating that cell-cell contacts are maintained. Interestingly, cells in the inner portion of the colony, which are organizing into rosettes, do not form lamellipodia and filopodia (Figure 4Bii, control, and Supplementary Movie 5), but do appear to spread basally. To quantify basal spreading during NPC-rosette formation, we assessed the basal membrane area (based on N-CADHERIN staining, Supplementary Figure 4A) of cells at the colony center at 1-hour (prior to rosette formation) and at 6-hour (the time of apical constriction initiation) time points (Figure 4D). These measurements were taken by confocal microscopy, focusing on the most basal surface. Interestingly, cells associated with forming rosettes at 6-hour show a clear increase in basal surface area. In contrast, central cells of the 6-hour colonies that are not associated with rosettes show a basal surface area that is similar to their basal area at 1 h (Figure 4D). Thus, rosette initiation is accompanied by basal spreading of the rosette-forming cells. This was not an artifact of cutting, since basal areas of cells forming spontaneous rosettes in H9 hESC-derived NPC monolayers (10 days 2Si treatment, Supplementary Figure 1A, top) are also significantly larger (Figure 4E) than the basal areas of non-rosette forming cells. Together, these results demonstrate that rapid basal expansion is an early step in rosette organization.

### Enhancement of Cell Spreading Increases Rosette Number, but Reduces Rosette Organization and Lumenal Size

Since basal spreading likely relies on the actin network of the cell, we next explored how modulating the activity of this network affects basal spreading and rosette formation. Lowering actomyosin contractility in an hPSC-derived neuroectoderm model was recently shown to expand cell area, leading to an increased colony area (Xue et al., 2018). Thus, we first tested the effect of further enhancing spreading, using Y-27632, a small molecule inhibitor of Rho-associated kinase, or ROCK (ROCK-i). Roller-dissociated colonies of 1196a hiPSC were allowed to attach for 2 h, then treated with ROCK-i. This treatment significantly increases colony area (Figures 4F,G), as expected. Interestingly, compared to controls, ROCK-i treatment leads to rosette formation in all colonies, and these colonies show an increased number of PODXL^+^ apical domains that maintain apical constriction as demonstrated by phosphorylated-MLC and closely spaced tight junctions (ZO-1) (Figures 4F,H,I and Supplementary Figure 4B). However, average lumenal size is decreased and cells surrounding the PODXL^+^ apical foci display significantly reduced radial organization (Figures 4J,K and Supplementary Movie 6), indicating that enhancing spreading with ROCK-i increases rosette initiation, but impairs NPC-radial morphogenesis and lumenal expansion.

### Colony Confinement Improves Rosette Organization

To further explore the role of active basal spreading in NPC rosette formation, we manipulated the degree of spreading by physically confining roller-dissociated segments on micropatterned surfaces of various sizes. To accomplish this, Geltrex solution (1%) was micro-printed onto otherwise non-stick PDMS surfaces. Three different sizes (small - 0.008 mm^2^, medium - 0.049 mm^2^ and large - 0.196 mm^2^) of Geltrex spots were tested; small and medium patterns were smaller than the average size of 12 h colonies from non-patterned conditions (0.124 mm^2^, Figure 4A). Plating cut colonies on these small and medium patterns limits the degree to which they can spread, while colonies can spread on larger patterns in an uninhibited manner. Notably, for all pattern sizes, the original size of the plated colony was the same, as determined by standard roller cutting of the 10-day monolayer. Thus, the relative number and density of cells initially plated on each Geltrex spot was the same for all pattern sizes.

**FIGURE 5:**
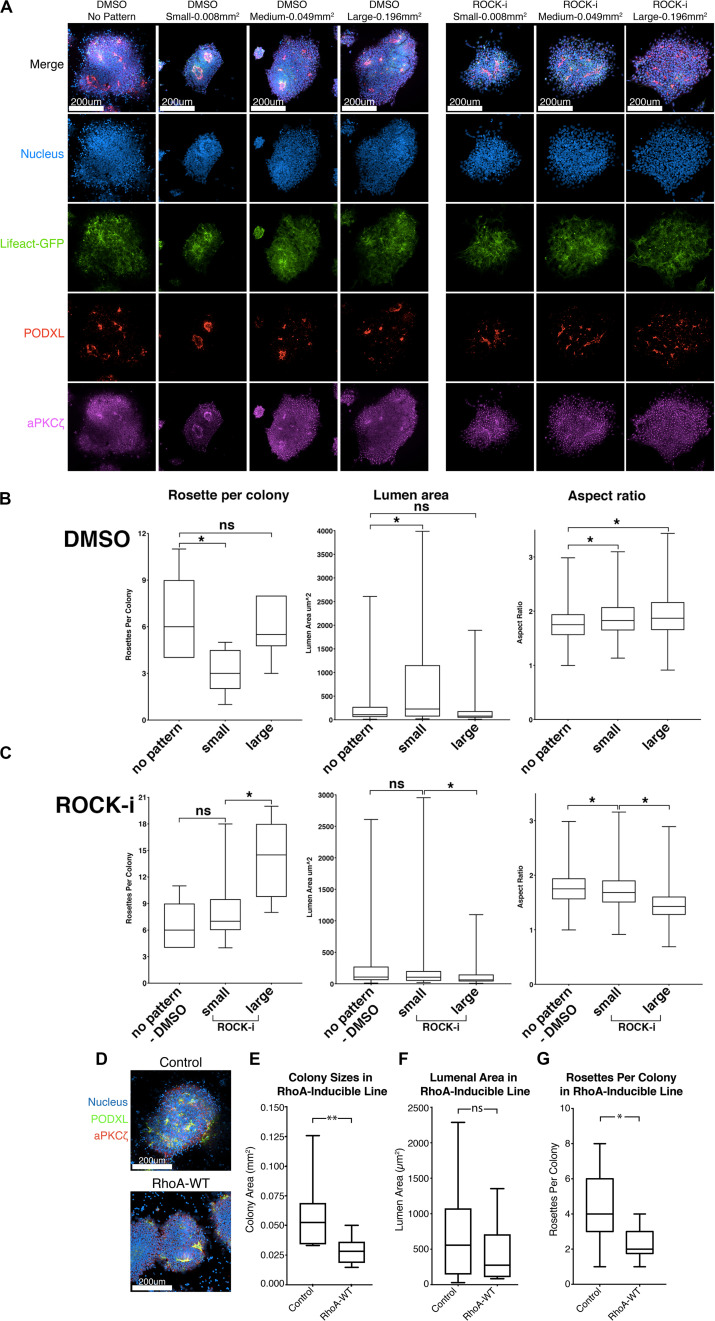
Effects of colony confinement on NPC rosette morphogenesis. **(A)** Confocal images of NPC rosettes, 12 h after culturing on un-patterned PDMS, or on small (0.008 mm^2^), medium (0.049 mm^2^) or large (0.196 mm^2^) circular patterns coated with 1% Geltrex in the absence (control, DMSO) or presence of ROCK-i, stained with indicated markers. **(B,C)** Quantitation of number of rosettes per colony (left), individual lumen area (middle) and nuclear aspect ratio (right) from DMSO (B) and ROCK-i **(C)** treated samples in panel **(A)**. Graphs containing all conditions are shown in Supplementary Figure 5. **(D)** Confocal images of control and RhoA-WT expressing NPC rosettes stained with apical markers, PODXL (green) and aPKCζ (red). **(E–G)** Quantitation of colony sizes **(E)**, individual lumen area **(F)**, and rosettes per colony **(G)** in control and RhoA-WT colonies. Scales as indicated. Student’s *t*-test was used for statistical analysis: ns = *p* > 0.05; *p ≤ 0.05; **p ≤ 0.01.

Marker analyses confirm that apical foci, surrounded by radially organized cells, are present in all colony sizes (Figure 5A). However, quantitation reveals that the number of apical foci per colony is significantly reduced in small patterns in which colony spreading is limited when compared to colonies grown on unpatterned Geltrex-coated substrates (Figure 5A, see DMSO group of rosette per colony in Figure 5B; and graphs including all conditions in Supplementary Figure 5). Colonies on the smallest patterns also exhibit a significant increase in lumenal size and slightly improved radial organization over the 12-hour culture period (see DMSO group in Figure 5B). Thus, confinement appears to reduce rosette initiation, but improve maturation of forming rosettes. This finding is in accordance with recently published results suggesting that plating neuronal progenitor cells on small micropatterns optimizes the formation of single, well-organized rosettes (Knight et al., 2018).

The data above suggest that colony confinement produces fewer but more well-organized rosettes, while enhancing colony spreading does the opposite. However, ROCK-i treatment likely perturbs other cellular processes in addition to its effect on spreading. Therefore, to specifically address the role of excessive spreading *per se* in the impaired radial organization phenotype, we tested whether colony confinement can rescue the phenotype imposed by ROCK-i (increased rosette number, but decreased lumen size and reduced radial organization). To do this, roller cut segments were cultured on small (0.008 mm^2^), medium (0.049 mm^2^) or large (0.196 mm^2^) circular patterns and treated with ROCK-i (Figure 5A). While colonies grown on large patterns show the full spectrum of the ROCK-i treatment phenotype, confining segments on small patterns rescues this phenotype: fewer PODXL^+^ apical foci are seen per colony, while radial organization and lumenal size are significantly increased, to levels similar to the “no pattern - DMSO” condition (Figures 5A,C, ROCK-i, compare to differences seen on non-patterned substrates, Figures 4I–K); data for all conditions are presented in Supplementary Figure 5). Together, these data suggest that basal cell spreading promotes rosette formation (apical foci per colony) and that the degree of spreading modulates overall NPC-rosette organization (lumenal area and radial organization).

### Genetic or Small Molecule-Induced Activation of Rho/ROCK Signaling Impairs Rosette Initiation

To further examine the relationship between cell spreading and rosette initiation, we inhibited spreading using a small molecule inhibitor as well as genetic perturbation. First, colonies were treated with 10 μM lysophosphatidic acid (LPA), an activator of Rho/ROCK signaling. Indeed, LPA-treated colonies fail to show signs of colony spreading, and NPC rosette morphogenesis is greatly reduced (Supplementary Figures 6A–E and Supplementary Movie 7). Next, to genetically test the influence of increased Rho signaling, we generated hiPSC carrying doxycycline-inducible RhoA. While, consistent with the LPA treatment, colony spreading is significantly reduced in cells treated with doxycycline and colonies exhibit reduced number of NPC rosettes per colony, RhoA-WT colonies show largely intact lumens demarcated by PODXL and aPKCξ, suggesting that increased RhoA activity does not completely disrupt NPC rosette morphogenesis (Figures 5D,E–G). These results confirm that active cell spreading is important for rosette initiation and point to an important role for proper levels of Rho/ROCK signaling in NPC rosette morphogenesis.

## Discussion

We report a simple and robust protocol that triggers the formation of NPC rosettes from hPSC-derived NPC monolayers within 6 h after induction. Using this system as a platform for highly efficient quantitative and mechanistic analyses, we demonstrate that Rho/ROCK-dependent basal NPC spreading promotes NPC-rosette initiation, and that NPC-rosette organization is modulated by the efficiency of basal spreading which also controls the size of NPC colonies.

NPC rosette morphogenesis is sensitive to culture conditions and is highly variable among hPSC lines; it is difficult to induce NPC rosette formation in some hPSC lines. In this study, we demonstrate the effects of different culture conditions on NPC rosette formation among multiple hPSC lines and establish an NPC rosette induction method that can robustly induce radial organization in hPSC lines that do not generally exhibit NPC rosette-forming property. We speculate that culture medium-based variability might be caused by differences in neural progenitor cell types; it will be of future interest to perform a systematic comparison of RNA expression profiles between hPSC grown in N2B27 supplements (Shi et al., 2012) vs. the mTeSR-based neural induction media used here. Furthermore, cellular mechanisms that control the propensity of hPSC to form NPC rosettes are not understood, though we as well as others have identified Rho/ROCK signaling as an important determinant (Knight et al., 2018). Since heterogeneity among hPSC lines has been recently noted (Rouhani et al., 2014; DeBoever et al., 2017; Kilpinen et al., 2017), a large-scale analysis using NPC derived from multiple hPSC lines with defined genetic backgrounds is needed to underpin variables that might control this propensity. Indeed, the 2Si/mTeSR-based NPC rosette induction protocol with roller-dissociation described here expands the ability of researchers to perform such mechanistic analyses in hPSC lines that could not have been used previously.

A major advantage of this protocol is its efficiency and reproducibility, as rosette organization is seen in the majority of colonies within 6 h after roller-dissociation, allowing for quantitative live imaging as well as small molecule, genetic and physical perturbations. Additionally, dozens of similarly sized colonies can be analyzed as independent units, permitting efficient statistical analyses of perturbations. Using these tools, we demonstrate that proper rosette formation requires basal spreading, ensured by inhibition of Rho/ROCK activity, while maturation of rosette organization (improved radial organization and lumenal expansion) is favored by confinement. Robust activation of Rho/ROCK, however, is detrimental to rosette formation, revealing a need for control of proper levels of signaling from this pathway. Importantly, detection of the phenomenon of colony spreading was enabled by high-resolution live cell imaging. Although Ziv et al. (2015) used live imaging to quantify nuclear movements within established mature rosettes, to our knowledge, ours is the first study to characterize morphological parameters surrounding NPC rosette initiation.

We provide strong evidence for a link between basally-driven cell spreading and apical organization of neural rosettes. Using live imaging, we show that basal cell spreading accompanies rosette organization. Physical confinement analyses, using circular micropatterned substrates combined with small molecule inhibitors, reveal that final neural rosette organization (apical foci per colony, lumen area and radial organization) is determined by the degree to which cells can spread. It will be of future interests to directly pursue whether cell spreading *per se*, or increased colony size (caused by spreading) is the most important factor for controlling NPC-rosette organization. Additionally, it is important to address whether rosette number within a colony affects rosette maturation and lumen formation since it might be speculated that when excess rosettes are present, fewer cells may be available for each rosette.

Interestingly, a recent study of rosette formation during *Drosophila* germ-band extension also concluded that basolaterally-driven cell movements precede the formation of apical rosette structures (Sun et al., 2017), similar to what we see in NPC rosettes. However, in that system, rosette morphogenesis is thought to be driven by planar cell polarity rather than apical constriction as in NPC, and basal protrusion activity is toward the rosette center rather than cell spreading toward the colony periphery. Nevertheless, basal cell movements appear to be key elements of this morphogenic process in both settings. These results show that proper apical organization of NPC rosettes is dependent on basal cell spreading, and that spreading must be tightly controlled to generate the single neural tube structure seen *in vivo*. It should also be noted that basal cell spreading may be a consequence of a previously described cell volume conservation mechanism involved in tissue morphogenesis (Gelbart et al., 2012), as reduction in apical surface area during apical construction must result in increased basal cell surface area (or *vice versa*) if cell volume is unchanged. These data also suggest a mechanistic explanation for the recent finding by Knight et al. (2018), that controlling the morphology of differentiating NPC colonies on micropatterns permits the generation of colonies with single rosettes and an organization that is more reminiscent of the *in vivo* neural tube.

We also confirm the need for Arp2/3 activity in rosette morphogenesis and show for the first time that the abundant neural sialoglycoprotein, PODXL, is necessary for lumenal expansion, as it is in MDCK cysts (Rodriguez-Boulan and Macara, 2014). PODXL, a transmembrane protein, has been shown to act both on the lumenal side, by forcing opposing membranes apart due to its heavy negative charge and on the cytosolic side, by affecting the apical actin network (Nielsen and McNagny, 2008). Indeed, the lumen in MDCK cysts arises by trafficking of apically charged vesicles to the cytokinetic plane and negatively charged PODXL is thought to be important in further expansion of the resulting lumen (Mangan et al., 2016; Mrozowska and Fukuda, 2016; Roman-Fernandez and Bryant, 2016). However, it is interesting to consider that NPC rosettes form in the context of a monolayer that is already polarized and they undergo lumen formation via apical constriction; thus PODXL may be utilized differently in NPC rosette formation vs. MDCK cysts. This remains to be tested. It will also be of interest to examine whether the previously described role of PODXL-dependent cell-cell adhesion is relevant to reduced lumenal expansion of rosettes in PODXL null cells (Takeda et al., 2000). Though the exact mechanism underlying the link between reduced or impaired PODXL activity and Juvenile Parkinsonism remains to be elucidated, we speculate that lumenal expansion during early neural tube morphogenesis may be impaired and that this in turn could perturb the forming neural epithelium. Consistent with this notion, *Podxl* deficient mouse hippocampal explants and primary hippocampal neurons display defective neurite outgrowth (Vitureira et al., 2010) and pan-neural *Podxl* loss produces malformation of ventricular spaces (Nowakowski et al., 2010).

Overall, the robust NPC rosette formation protocol described here, combined with a micropattern-based confinement method to generate NPC rosettes, provides a novel *in vitro* avenue to perform mechanistic analyses of rosette morphogenesis using neural progenitor cells derived from hESC or hiPSC.

## Materials and Methods

### Stem Cell Maintenance and Passaging

Human hPSC used in this work included H9 hESC (WA09) as well as 1196a and 20-1 iPSC. All protocols for the use of the hPSC lines were approved by the Human Pluripotent Stem Cell Research Oversight Committee at the University of Michigan. The hESC and hiPSC lines were cultured and maintained as described (Ludwig et al., 2006), using lactate dehydrogenase-elevating virus (LDEV)-free hESC-qualified Geltrex (Thermo Fisher, derived from reduced growth factor ECM, extracted from murine Engelbreth-Holm-Swarm sarcoma cells similar to Matrigel) to prepare ECM-coated plates. All cell lines tested negative for mycoplasma contamination (LookOut Mycoplasma PCD Detection kit; Sigma-Aldrich).

### Neural Rosette Induction

Cells cultured in maintenance conditions were disassociated using 1 mL of Accutase (Sigma-Aldrich) and incubated at 37°C for 10 min. Cells were agitated thoroughly with a P1000 pipetman to ensure complete dissociation, then resuspended in DMEM-F12 and spun down to pellet. The supernatant was removed and cells were resuspended in mTeSR containing 10 μM Y-27632, a ROCK inhibitor necessary to inhibit dissociation mediated apoptosis of hPSC (Watanabe et al., 2007). Cells were plated at a density of 6.67 × 10^4^ cells per cm^2^ or approximately 600,000 cells per well of a 6-well plate in 1% Geltrex coated wells and incubated for 24 h. Y-27632 was then removed by washing with DMEM;F12 and cells were incubated in mTeSR containing 10 μM SB-431542 and 0.5 μM LDN-193189 (2Si). Cells were maintained in mTeSR/2Si for 9 days with daily medium change. A tightly packed monolayer appeared by the end of day 10. A StemPro^®^ EZPassage^TM^ Disposable Stem Cell Passaging Tool was used to cut the monolayer into 0.022 mm^2^ sections and these sections were removed by scraping (roller-dissociation). Cut and scraped sections were re-plated onto a glass coverslip coated with 1% Geltrex. Rosettes began to appear 6 h after replating, and continued to mature for approximately 12 h. Samples were fixed using 4% PFA for 30 min, then washed with PBS solution prior to immunostaining. For micropattern assays, the roller-cut colonies in mTeSR/2Si were detached from substrates by scraping, which were re-plated onto micropatterned substrates. After 2 h, colony attachment to substrates was confirmed and unattached colonies were washed off (3× washes using DMEM;F12). Medium (mTeSR/2Si) was added for an additional 10 h. This method reproducibly allows for the attachment of a single colony per micropattern. Since colonies are of similar size, this means that all micropatterns contain approximately the same number of plated cells. For ROCK inhibition (Y-27632) and Rho activation assays (LPA), ROCK-i was added 2 h after plating.

### Immunostaining and Characterization

Assays were induced to make neural rosettes as described above and fixed at varying time points from 6 to 72 h. Antibodies for immunofluorescence staining were: anti-Nestin (1:100, Santa-Cruz Biotechnologies, sc-23927), anti-PKC-ζ (1:250, sc-216, Santa-Cruz), N-cadherin (1:500, MNCD2-c, DSHB), anti-Podxl (1:2000, MAB1658, R&D Systems), anti-Sox2 (1:1000, 09-0024, Stemgent), anti-Pax3 (1:200, R&D Systems, NBP1-32944), anti-Rab11 (1:500, #610656, BD), E-cadherin (1:500, #610182, BD), anti-GM130 (1:500, #610822, BD), Arl13B (1:500, 17711-1-AP, Proteintech), ZO-1 (1:200, sc-33725, Santa-Cruz Biotechnologies) phosphorylated myosin light chain (Ser19) (1:400, 3671S, Cell Signaling Technologies). DNA and membrane was labeled using Hoechst 33258 (Life Technologies) and wheat germ agglutinin (1:250, Life Technologies), respectively. Goat-raised secondary antibodies labeled with various fluorophores (1:500, Life Technologies) were used. Imaging was done using a Nikon A-1 confocal microscope, and images were analyzed and generated using Imaris (Bitplane), Photoshop CS6 (Adobe) or ImageJ (National Institute of Health). The free hand tool on ImageJ was used to outline lumenal area demarcated by anti-PODXL immunostaining and total pixel area was measured. Aspect ratio was quantified by measuring the perpendicular length of the nuclei bordering the apical surface of the rosette and dividing this by the parallel width of the nuclei.

### Small Molecule Drug Inhibitor Assays

Colonies were cultured with or without indicated small molecule modulators, CK-666 (500 μM, Arp2/3 inhibitor, EMD Millipore), Y-27632 (10 μM, Rho inhibitor, Stem Cell Technologies) and lysophosphatidic acid (Rho activator, Cayman). DMSO was added in control groups. The amount of DMSO added to the experimental condition is the same as that of added to the control condition.

### Microcontact Printing

We used a two-step micropatterning technique using a standard microcontact printing process to pattern ECM protein on the substrate and then recoated the patterned substrate with 1% Geltrex to improve protein attachment. For microcontact printing, patterned stamps were generated using replica molding from a silicon mold fabricated by standard photolithography and deep reactive ion etching (DRIE) (Fu et al., 2010). A flat 1:15 PDMS stamp was prepared and inked with Geltrex for 24 h at 4°C to absorb protein via hydrophilic interactions between the stamp and the Geltrex. The PDMS stamp was then thoroughly rinsed with distilled water and blown dry with a stream of nitrogen. In parallel, the PDMS coated coverslips were treated with ultraviolet (UV) ozone (UV-ozone cleaner; Jelight, Irvine, CA, United States) for 7 min to oxidize the PDMS surface and change the PDMS surface from hydrophobic to hydrophilic, allowing complete transfer of Geltrex from the PDMS stamp to the coverslip. The PDMS stamp was placed in contact with the coverslip for about 5 s to complete the Geltrex transfer process. To avoid protein adsorption to non-functionalized regions of the coverslip, the coverslip surface was treated with pluronics F127 NF dissolved in PBS (0.2%, w/v; BASF, Ludwigshafen, Germany) for 30 min at room temperature and then washed three times with distilled water. To recoat the patterned substrate with Geltrex, coverslips patterned by microcontact printing were first immersed in mTeSR for at least 2 h to prevent protein adsorption to PDMS surfaces not coated with Geltrex and then recoated with 1% Geltrex for an additional hour. The substrate was then washed with PBS several times to remove any protein attached to unpatterned regions (Nasr Esfahani et al., 2019).

### Live Imaging

Lifeact- or PODXL-GFP expressing 1196a hiPSC (see cloning in the Constructs and cell lines section) were plated on six-well plates (Nunc) in mTeSR1/2Si medium, and time-lapse images were taken at 37°C using the IncuCyte Zoom live cell imaging (Sartorius). Alternatively, cells were plated on a glass bottom culture dish (MatTek), and were imaged in a live-cell imaging chamber (Tokai HIT) configured for Olympus IX-83 at 37°C.

### Constructs and Cell Lines

The piggyBac transposon system was used to prepare cell lines expressing PODXL-EGFP and Lifeact-EGFP using a procedure previously described (Taniguchi et al., 2015). PODXL-EGFP constructs have been described previously (Taniguchi et al., 2017). Lifeact-EGFP (46356; Addgene; Iain Cheeseman) was PCR amplified (forward: 5′-GCGAATTCGCCACCATGGGTGTCGCAG-3′; reverse: 5′-CGGCGGCCGCTTACTTGTACA GCTCGTC-3′). The amplified product was then subcloned into the pPBCAG-GFP piggyBac transposon vector (Chen and LoTurco, 2012) which was digested with *EcoRI* and *NotI*. Transfection was done as previously described (Taniguchi et al., 2015). Fluorescence-activated cell sorting was performed to collect cells stably expressing specific fluorophores. Inducible expression of RhoA-EGFP constructs was performed using an Inducible All-in-One piggyBac Transposon System (Kim et al., 2016). RhoA-WT and –CA constructs (12965 and 12968; Addgene) (Subauste et al., 2000) were PCR amplified (forward: 5′- CACC-GCCACC-ATGGTGAGCAAGGGC-3′; reverse: 5′- TCACAAGACAAGGCACCC-3′) and subcloned into pENTR-dTOPO (Thermo Fisher). Lastly, using the Gateway L-R clonase II system (Invitrogen), RhoA constructs were subcloned into the PB-TA-ERP2 piggyBac All-in-One inducible destination vector (80477; Addgene) (Kim et al., 2016).

### Genome Editing for *PODXL* Knockout

*PODXL* knockout 1196a hiPSC were generated using a piggyBac transposon-CRISPR/Cas9-based genome editing construct as previously described (Shao et al., 2017b). To introduce insertion/deletion (indel) mutations into exon 1 of the human *PODXL* locus (as shown in Supplementary Figure 2) via non-homologous end joining (NHEJ), a gRNA targeting sequence (GCGTCGAAGTGGGTTGTCGG) was designed (using published algorithms)^1^. The annealed oligo containing the gRNA sequence (sense: 5′- CACCGGCGTCGAAGTGGGTTGTCGG-3′; anti-sense: 5′- AAACCCGACAACCCACTTCGACGCC-3′) was subcloned into *BbsI* sites to generate the piggyBac-CRISPR/Cas9 construct containing the *PODXL* gRNA targeting sequence (piggyBac-CRISPR/Cas9-*PODXL*). This construct was transfected into 1196a hiPSC, sorted for GFP^+^ cells using fluorescence-activated cell sorting (FACS). Resulting clonally purified cells were genotyped as previously described (Shao et al., 2017b). In control cells, a piggyBac-CRISPR/Cas9 vector lacking the gRNA targeting sequence was used.

### RNA Isolation and RT-PCR

RNA was extracted from hiPSC monolayer cultures, at 10d of neural induction (immediately before roller-dissociation), or 24 h after roller-dissociation, using RNeasy Micro Kit (Qiagen). RNA quality and quantity were determined spectrophotometrically using NanoDrop 2000 (Thermo). Reverse transcription was conducted using the SuperScript VILO kit (Life Technologies). qRTPCR was performed using Quantitect Sybr Green MasterMix (Qiagen) on a Step One Plus Real-Time PCR system (Life Technologies). Primers specific to *OCT4*, *NANOG*, *OTX1*, *PAX3*, *PAX6*, *ZBTB16*, *LMO3*, NR2F1, *PLAGL1*, *LIX1*, *EVI1*, and *DACH1* were used. Resulting values were then normalized to GAPDH. A standard curve of GAPDH control with 90% efficiency was generated to determine relative abundance of tested messages.

### List of RT-PCR Primers

**Table:** 

h-Otx1-F	GCGTCGTCGCTGAGTACAC
h-Otx1-R	ACATGGGATAAGAGGCTGCTG
h-Pax3-F	AGCTCGGCGGTGTTTTTATCA
h-Pax3-R	CTGCACAGGATCTTGGAGACG
h-Pax6-F	TGGGCAGGTATTACGAGACTG
h-Pax6-R	ACTCCCGCTTATACTGGGCTA
h-Tuj1-F (TUBB3)	GGCCAAGGGTCACTACACG
h-Tuj1-R (TUBB3)	GCAGTCGCAGTTTTCACACTC
h-Dach1-F	ATGTGGAACAAGTTCGCATCC
h-Dach1-R	TGCAGTCATTGTAGAGGGTCT
h-ZBTB16-F (PLZF)	GAGATCCTCTTCCACCGCAAT
h-ZBTB16-R (PLZF)	CCGCATACAGCAGGTCATC
h-LMO3-F	GACACCAAGCCGAAAGGTTG
h-LMO3-R	ATGCCAGTATTTGTCCAGTGC
h-PLAGL1-F	AAAGATGCTTCTACACCCGGA
h-PLAGL1-R	AGTGGGTCTTCTTGGTATGCC
h-Lix1-F	CACAGAGATCCGGCTCTAGTC
h-Lix1-R	CACGTAACTCACAAAGGGAGG
h-Evi1-F (MECOM)	TATCCACGAAGAACGGCAATATC
h-Evi1-R (MECOM)	CATGGAAACTTTTGGTGATCTGC
h OCT4 F	GTGGAGGAAGCTGACAACAA
h OCT4 R	GGTTCTCGATACTGGTTCGC
h NANOG F	GATTTGTGGGCCTGAAGAAA
h NANOG R	ATGGAGGAGGGAAGAGGAGA
hGAPDH-F	CTCTGCTCCTCCTGTTCGAC
hGAPDH-R	TTAAAAGCAGCCCTGGTGAC

## Data Availability Statement

The raw data supporting the conclusions of this article will be made available by the authors, without undue reservation.

## Author Contributions

RT, YS, DG, and KT designed the experiments. RT, YS, SW, CC, and KT performed the experiments. JS established the 20-1 hiPSC line. KO’S derived the 1196a hiPSC line. YS, SN, and JF designed the Geltrex-ECM micropattern system. RT, YS, DG, and KT analyzed the data and wrote the manuscript. All authors read and edited the manuscript.

## Conflict of Interest

The authors declare that the research was conducted in the absence of any commercial or financial relationships that could be construed as a potential conflict of interest.

## Footnotes

1 http://crispr.mit.edu

## Supplementary Material

Supplementary Figure 1 **(A)** H9 hESC, 1196a hiPSC or 20-1 hiPSC, cultured in mTeSR/2Si neural differentiation media, were examined in 10-day (d10) monolayers, prior to dissociation (left, 10 day), or were examined 24 h after roller-dissociation and re-plating of d10 monolayers (right, 24-hour colony). Cells were stained for apical markers, PODXL (green) and aPKCζ (red). X-Z planes reveal polarized monolayers. **(B)** 1196a hiPSC cultured in E6/2Si and examined in a d10 monolayer. Cells were apically polarized as shown by staining for apical markers, PODXL (green) and aPKCζ (red). **(C)** H9 hESC and 1196a hiPSC, cultured in neural differentiation conditions outlined by Shi et al. (2012) were harvested on d10, prior to dissociation (left, 10 day), or were harvested 24 h after roller-dissociation and re-plating of d10 monolayers (right, 24-hour colony). Cells were stained for indicated apical markers. X–Z planes reveal polarized monolayer morphology. While PODXL^+^/membrane^+^ foci are seen using 1196a hiPSC (24-hour colony) using a method described by Shi et al., these foci are not surrounded by radially organized NPC (see “i”). **(D)** Confocal micrographs of roller-cut (left) and manually dissociated (right) colonies stained with indicated markers, 1 h after plating. **(E)** Schematic of the roller-based StemPro^®^ EZPassage^TM^ Disposable Stem Cell Passaging Tool. **(F)** Colony size quantitation of roller-cut and scraped colonies. The roller-cut method shows significantly less variability in colony size (*F*-test: *p* < 0.05). **(G)** Wide-field confocal images of roller-dissociated colonies 6 h after plating, stained with indicated markers, revealing the formation of multiple aPKCζ^+^ apical foci throughout the colony. **(H)** Confocal images of three representative colonies 12 h after roller-cutting without replating. Orange dotted boxes indicate the size of colonies after roller-cut. White dotted boxes indicate the edge of colonies after expansion. No PODXL^+^ foci were seen, even when neighboring colonies were removed to provide additional space to spread (middle, right panels). **(I)** Immunolocalization of TUJ1, a neuron-specific Class III β-tubulin, in a rosette generated from cells carrying Lifeact-GFP, cultured for 8 days after roller-dissociation. Wide-field image is shown to reveal the formation of abundant TUJ1^+^ rosettes.

Supplementary Figure 2 **(A–F)** Confocal images of NPC colonies treated with DMSO (control) and 500 μM CK-666 for 10 h after colonies were allowed to attach for 2 h **(A)**. A representative image of NPC-rosettes in control samples is used to show how nuclear aspect ratio (nuclear length per width, dotted white cross, (i) and lumenal area (dotted white shape, (ii) are measured **(B)**. Upon CK-666 treatment, significant reduction in lumenal area and nuclear aspect ratio are seen **(C,D)**, while colony size and number of rosettes per colony are not significantly different **(E,F)**. Scales as indicated. Student’s *t*-test was used for statistical analysis: ^∗^*p* ≤ 0.05.

Supplementary Figure 3 **(A)** The sequence of human *PODXL* exon 2. gRNA target and PAM sequence are shown in red and green, respectively. **(B)** The edited sequence of *PODXL*-KO #1 (middle) and *PODXL*-KO #2 (bottom) are shown with respect to the *PODXL*-wild-type sequence. **(C)** Chromatographs for edited alleles are shown. Red underscore – target sequence after editing; green underscore – PAM sequence after editing; red box – inserted sequence.

Supplementary Figure 4 **(A)** A representative confocal micrograph near the basal surface of cells stained for N-CADHERIN in control samples is used to show how basal cell area (dotted white shape) was measured. **(B)** Confocal images of DMSO and ROCK-i treated samples 12 h after roller-dissociation stained with indicated markers. Some degree of apical constriction (p-MLC) as well as tight junction (ZO-1) formation was maintained in ROCK-i samples. **(C)** Immunolocalization analysis of p-MLC in colony edge of DMSO and ROCK-i treated samples, 12 h after roller-dissociation. Scales as indicated.

Supplementary Figure 5 **(A–C)** Quantitation [rosette per colony **(A)**, lumenal area **(B)** and nuclear aspect ratio **(C)**] in all conditions in Figure 5A.

Supplementary Figure 6 **(A)** Confocal images of NPC rosettes treated with DMSO (control), ROCK-i or LPA, stained with indicated markers. **(B–E)** Quantitation for colony size **(B)**, number of rosettes per colony **(C)**, lumenal area **(D)**, and nuclear aspect ratio **(E)** from DMSO (control), ROCK-i and LPA treated samples in panel **(A)**. Control and ROCK-i images in panel **(A)** are from Figure 4G. Control and ROCK-i quantitation data in panels **(B–E)** are identical to Figures 4H–L. Scales as indicated. Student’s *t*-test was used for statistical analysis: ^∗^*p* ≤ 0.05; ^∗∗^*p* ≤ 0.01; and ^∗∗∗^*p* ≤ 0.001.

Supplementary Movie 1 Live imaging of roller-cut 1196a Lifeact-GFP colonies. Imaging was started 2 h after roller-dissociating the d10 NPC monolayer.

Supplementary Movie 2 Live imaging of roller-cut 1196a colonies expressing PODXL-GFP. Imaging was started 2 h after roller-dissociating the d10 NPC monolayer.

Supplementary Movie 3 Live imaging of colony spreading of 1196a hiPSC colony stably expressing Lifeact-GFP. Imaging was started 5 h after roller-dissociation of the d10 NPC monolayer.

Supplementary Movie 4 Live imaging of colony edge (as shown in Figure 4Bi, control) during spreading of 1196a Lifeact-GFP hiPSC in Supplementary Movie 3.

Supplementary Movie 5 Live imaging of colony center (as shown in Figure 4Bii, control) during spreading of 1196a Lifeact-GFP hiPSC in Supplementary Movie 3.

Supplementary Movie 6 Live imaging of roller-cut 1196a PODXL-GFP colonies treated with 10 μM ROCK-i (Y-27632). Imaging and ROCK-i treatment were initiated 2 h after roller-dissociation of the d10 NPC monolayer.

Supplementary Movie 7 Live imaging of roller-cut 1196a PODXL-GFP colonies treated with 10 μM LPA (lysophosphatidic acid). Imaging and LPA treatment were initiated 2 h after roller-dissociation of the d10 NPC monolayer.
