# Supplementary material for: Effect of Cell Spreading on Rosette Formation by Human Pluripotent Stem Cell-Derived Neural Progenitor Cells
Source: Front Cell Dev Biol. 2020 Oct 15;8:588941. doi: 10.3389/fcell.2020.588941 (PMC7593581; doi:10.3389/fcell.2020.588941)
Supplement: Supplementary Figure 1 — (A) H9 hESC, 1196a hiPSC or 20-1 hiPSC, cultured in mTeSR/2Si neural differentiation media, were examined in 10-day (d10) monolayers, prior to dissociation (left, 10 day), or were examined 24 h after roller-dissociation and re-plating of d10 monolayers (right, 24-hour colony). Cells were stained for apical markers, PODXL (green) and aPKCζ (red). X-Z planes reveal polarized monolayers. (B) 1196a hiPSC cultured in E6/2Si and examined in a d10 monolayer. Cells were apically polarized as shown by staining for apical markers, PODXL (green) and aPKCζ (red). (C) H9 hESC and 1196a hiPSC, cultured in neural differentiation conditions outlined by Shi et al. (2012) were harvested on d10, prior to dissociation (left, 10 day), or were harvested 24 h after roller-dissociation and re-plating of d10 monolayers (right, 24-hour colony). Cells were stained for indicated apical markers. X–Z planes reveal polarized monolayer morphology. While PODXL+/membrane+ foci are seen using 1196a hiPSC (24-hour colony) using a method described by Shi et al., these foci are not surrounded by radially organized NPC (see “i”). (D) Confocal micrographs of roller-cut (left) and manually dissociated (right) colonies stained with indicated markers, 1 h after plating. (E) Schematic of the roller-based StemPro® EZPassageTM Disposable Stem Cell Passaging Tool. (F) Colony size quantitation of roller-cut and scraped colonies. The roller-cut method shows significantly less variability in colony size (F-test: p < 0.05). (G) Wide-field confocal images of roller-dissociated colonies 6 h after plating, stained with indicated markers, revealing the formation of multiple aPKCζ+ apical foci throughout the colony. (H) Confocal images of three representative colonies 12 h after roller-cutting without replating. Orange dotted boxes indicate the size of colonies after roller-cut. White dotted boxes indicate the edge of colonies after expansion. No PODXL+ foci were seen, even when neighboring colonies were removed to provi [file Image_1.pdf]

Supplementary Figures

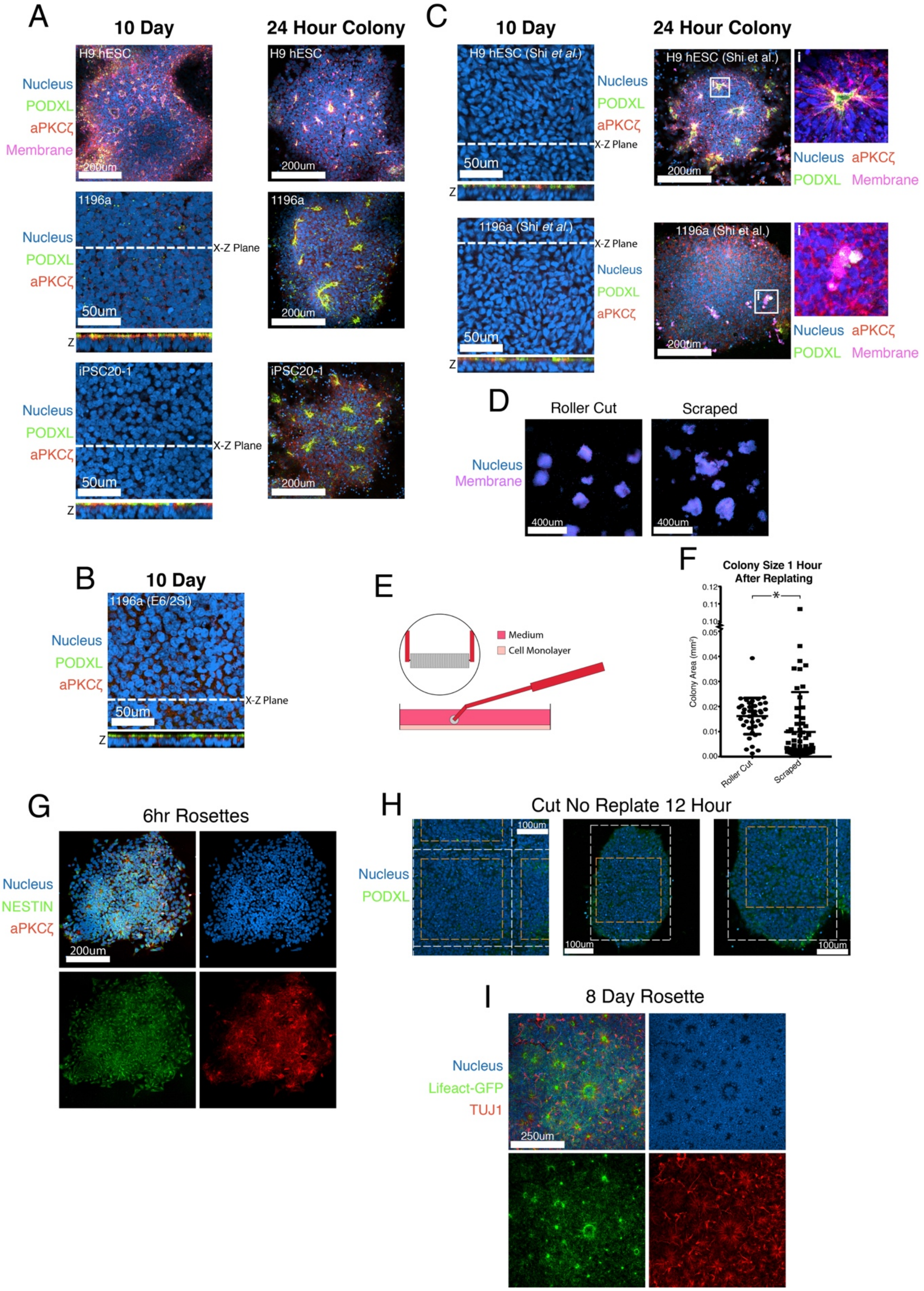

Supplementary Figure 1. A) H9 hESC, 1196a hiPSC or 20-1 hiPSC, cultured in mTeSR/2Si neural differentiation media, were examined in 10-day (d10) monolayers, prior to dissociation (left, 10 day), or were examined 24 hours after roller-dissociation and re-plating of d10 monolayers (right, 24 hour colony). Cells were stained for apical markers, PODXL (green) and aPKCζ (red). X-Z planes reveal polarized monolayers. B) 1196a hiPSC cultured in E6/2Si and examined in a d10 monolayer. Cells were apically polarized as shown by staining for apical markers, PODXL (green) and aPKCζ (red). C) H9 hESC and 1196a hiPSC, cultured in neural differentiation conditions outlined by Shi *et al.* (Shi et al., 2012) were harvested on d10, prior to dissociation (left, 10 day), or were harvested 24 hours after roller-dissociation and re-plating of d10 monolayers (right, 24-hour colony). Cells were stained for indicated apical markers. X-Z planes reveal polarized monolayer morphology. While PODXL$^+$/membrane$^+$ foci are seen using 1196a hiPSC (24 hour colony) using a method described by Shi *et al*., these foci are not surrounded by radially organized NPC (see “i”). D) Confocal micrographs of roller-cut (left) and manually dissociated (right) colonies stained with indicated markers, 1 hour after plating. E) Schematic of the roller-based StemPro® EZPassage™ Disposable Stem Cell Passaging Tool. F) Colony size quantitation of roller-cut and scraped colonies. The roller-cut method shows significantly less variability in colony size (F-test: $p<0.05$). G) Wide-field confocal images of roller-dissociated colonies 6 hours after plating, stained with indicated markers, revealing the formation of multiple aPKCζ^+ apical foci throughout the colony. H) Confocal images of three representative colonies 12 hours after roller-cutting without replating. Orange dotted boxes indicate the size of colonies after roller-cut. White dotted boxes indicate the edge of colonies after expansion. No PODXL$^+$ foci were seen, even when neighboring colonies were removed to provide additional space to spread (middle, right panels). I) Immunolocalization of TUJ1, a neuron-specific Class III α-tubulin, in a rosette generated from cells carrying Lifeact-GFP, cultured for 8 days after roller-dissociation. Wide-field image is shown to reveal the formation of abundant TUJ1$^+$ rosettes.

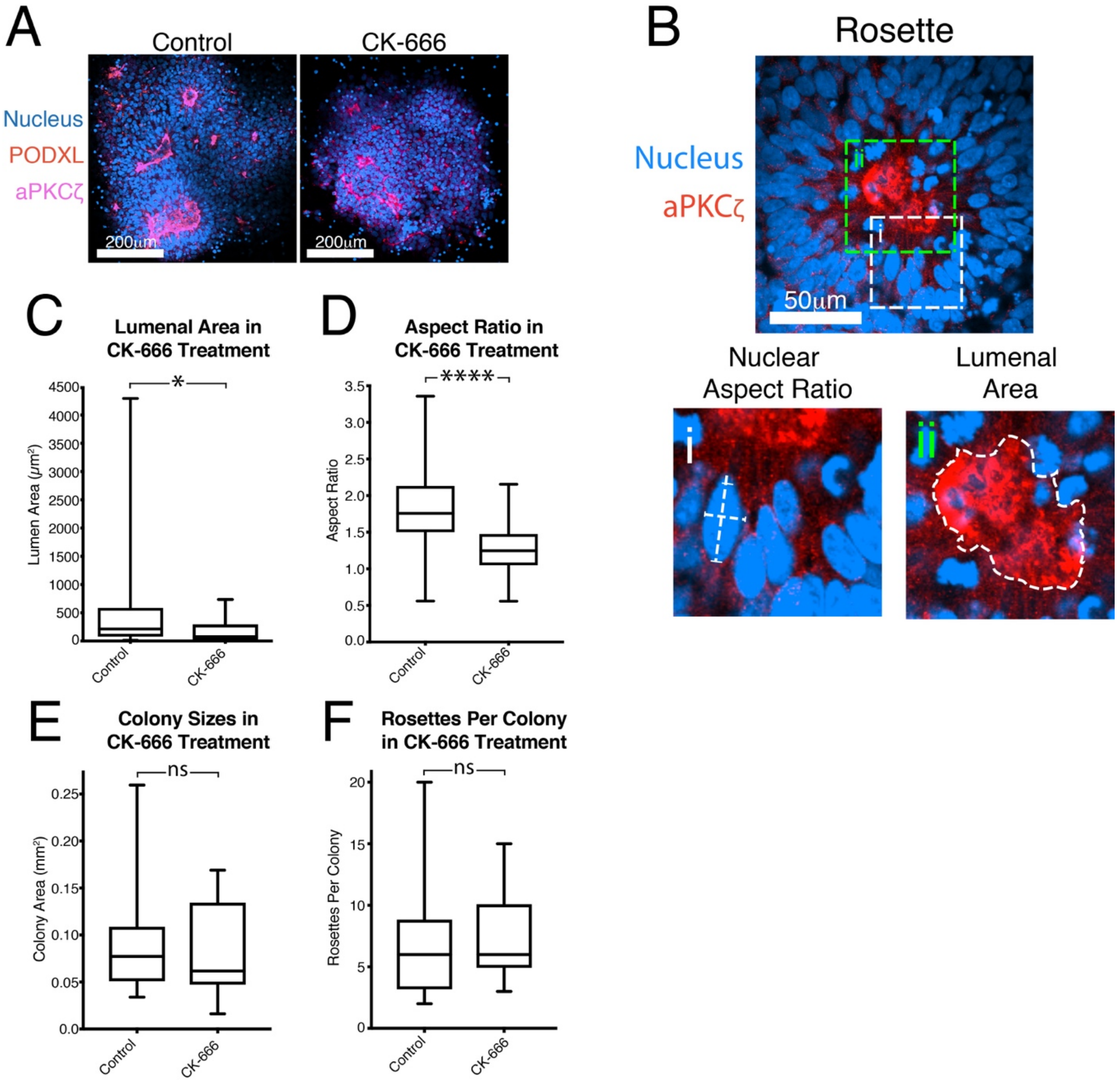

Supplementary Figure 2. A-F) Confocal images of NPC colonies treated with DMSO (control) and 500μM CK-666 for 10 hours after colonies were allowed to attach for 2 hours (A). A representative image of NPC-rosettes in control samples is used to show how nuclear aspect ratio (nuclear length per width, dotted white cross, i) and lumenal area (dotted white shape, ii) are measured (B). Upon CK-666 treatment, significant reduction in lumenal area and nuclear aspect ratio are seen (C,D), while colony size and number of rosettes per colony are not significantly different (E,F).
Scales as indicated. Student's t-test was used for statistical analysis: * = $p \leq 0.05$.

A

human *PODXL* CDS1

CAACCCAGACTACTACGGACTCATCTAACAAAACAGCACCGACTCCAGCATCCAGTGTCACCATCATGGCTACAGATACAGCCC
AGCAGAGCACAGTCCCCACTTCCAAGGCCAACGAAATCTTGGCCTCGGTCAAGGCGACCACCCTTGGTGTATCCAGTGACTCA
CCGGGGACTACAACCCTGGCTCAGCAAGTCTCAGGCCCAGTCAACACTACCGTGGCTAGAGGAGGCGGCTCAGGCAACCCTA
CTACCACCATCGAGAGCCCCAAGAGCACAAAAAGTGCAGACACCACTACAGTTGCAACCTCCACAGCCACAGCTAAACCTAAC
ACCACAAGCAGCCAGAATGGAGCAGAAGATACAACAAACTCTGGGGGGAAAAGCAGCCACAGTGTGACCACAGACCTCACATC
CACTAAGGCAGAACATCTGACGACCCCTCACCCTACAAGTCCACTTAGCCCCCGACAACCCACTTTGACGCATCCTGTGGCCA
CCCCAACAAGCTCGGGACATGACCATCTTATGAAAATTTCAAGCAGTTCAAGCACTGTGGCTATCCCTGGCTACACCTTCACAAG
CCCGGGGATGACCACCACCCTAC

B

(Green: PAM Red: target sequence)

PODXL -WT 1. AC-----TTAGCCCCCGACAACCCACTTTGACGCATCCT
2. AC-----TTAGCCCCCGACAACCCACTTTGACGCATCCT

PODXL -KO #1 1. AC-----------------------TTTGACGCATCCT (19 bp deletion)
2. ACCCTACAAGTCCACTTACAACCCACTTTGACGCATCCT (10 bp deletion with 15 bp insertion)

PODXL -KO #2 1. AC-----TTAGCCCCC-----------TTTGACGCATCCT (10 bp deletion)
2. AC-----TTAGC----TACAACCCACTTTGACGCATCCT (5 bp deletion with 1 bp insertion)

C

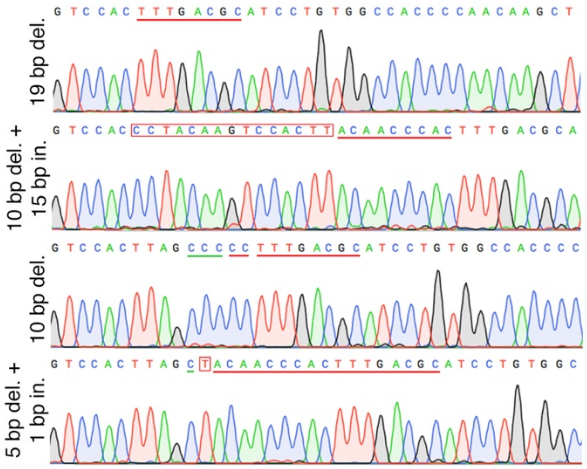

Supplementary Figure 3. A) The sequence of human *PODXL* exon 2. gRNA target and PAM sequence are shown in red and green, respectively. B) The edited sequence of *PODXL*-KO #1 (middle) and *PODXL*-KO #2 (bottom) are shown with respect to the *PODXL*-wild-type sequence. C) Chromatographs for edited alleles are shown. Red underscore – target sequence after editing; green underscore – PAM sequence after editing; red box – inserted sequence.

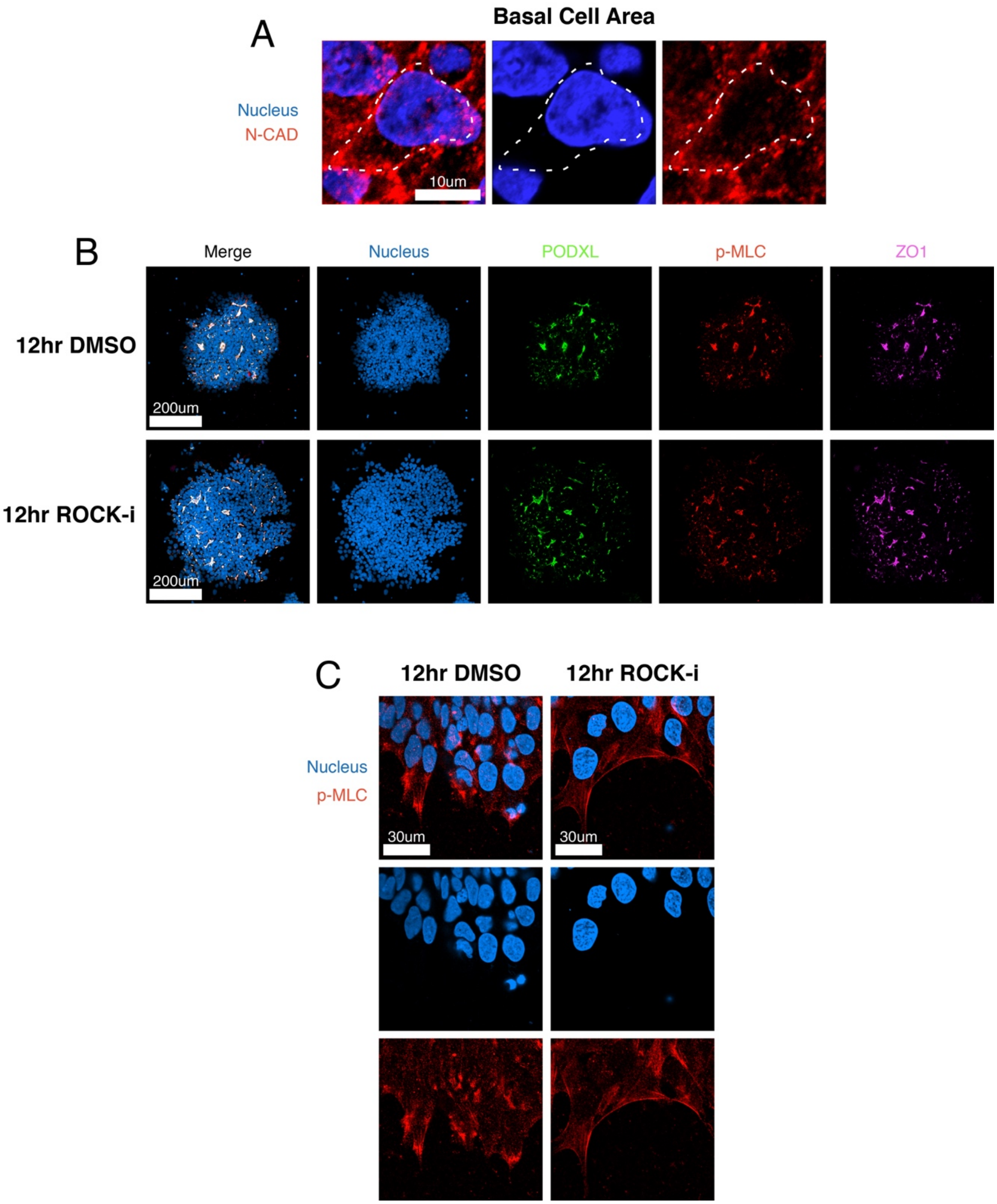

Supplementary Figure 4. A) A representative confocal micrograph near the basal surface of cells stained for N-CADHERIN in control samples is used to show how basal cell area (dotted white shape) was measured. B) Confocal images of DMSO and ROCK-i treated samples 12 hours after roller-dissociation stained with indicated markers. Some degree of apical constriction (p-MLC) as well as tight junction (ZO-1) formation was maintained in ROCK-i samples. C) Immunolocalization analysis of p-MLC in colony edge of DMSO and ROCK-i treated samples, 12 hours after roller-dissociation. Scales as indicated.

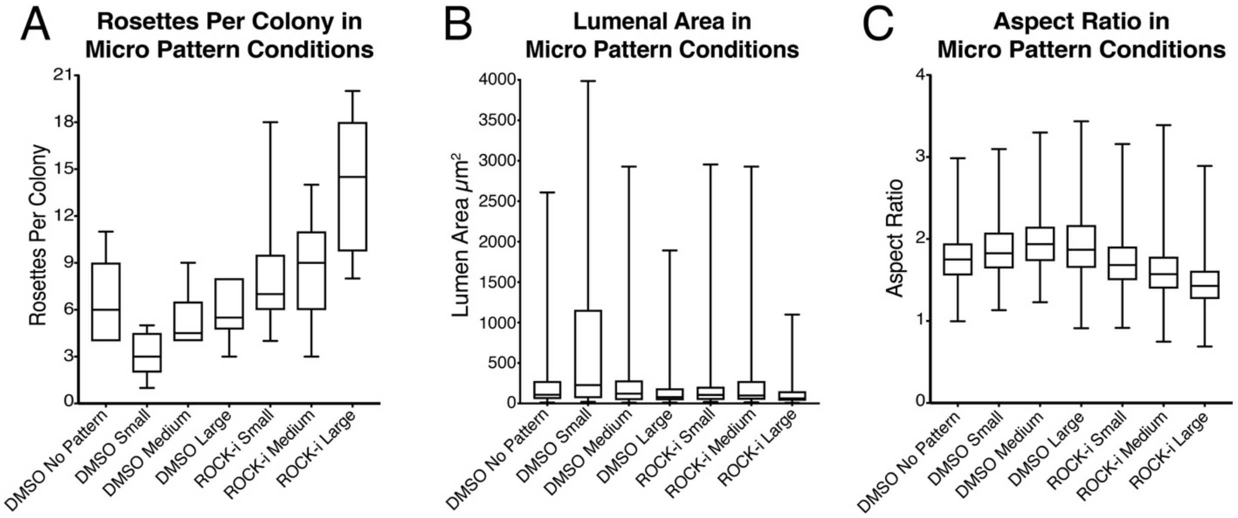

Supplementary Figure 5. A-C) Quantitation (rosette per colony (A), lumenal area (B) and nuclear aspect ratio (C)) in all conditions in Fig. 5A.

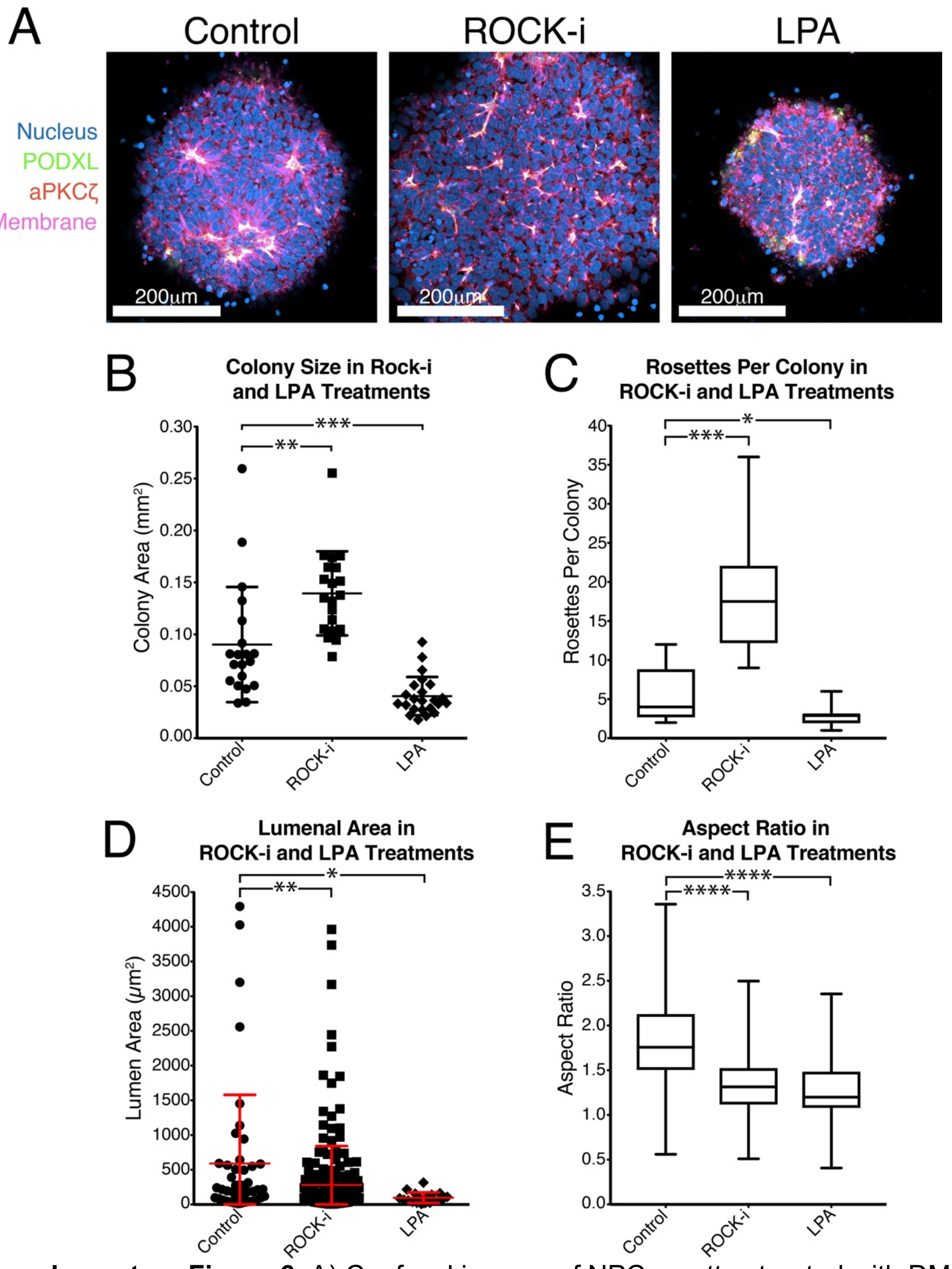

Supplementary Figure 6. A) Confocal images of NPC rosettes treated with DMSO (control), ROCK-i or LPA, stained with indicated markers. B-E) Quantitation for colony size (B), number of rosettes per colony (C), lumenal area (D) and nuclear aspect ratio (E) from DMSO (control), ROCK-i and LPA treated samples in (A). Control and ROCK-i images in (A) are from Fig. 4G. Control and ROCK-i quantitation data in (B-E) are identical to Fig. 4H-L. Scales as indicated. Student's t-test was used for statistical analysis: * = $p \leq 0.05$; ** = $p \leq 0.01$ and *** = $p \leq 0.001$.
